# Scientific quality of colonoscopy-related YouTube videos in the Turkish language

**DOI:** 10.1097/MD.0000000000048231

**Published:** 2026-04-03

**Authors:** Ibrahimhalil Dusunceli, Zeynep Gök Sargin, Fatih Sargin

**Affiliations:** a Department of Gastroenterology and Hepatology, Faculty of Medicine, Zonguldak Bulent Ecevit University, Zonguldak, Türkiye; b Department of Gastroenterology, Antalya City Hospital, Antalya, Türkiye; c Department of Critical Care, Antalya City Hospital, Antalya, Turkey.

**Keywords:** colonoscopy, quality, Turkish language, YouTube

## Abstract

Colonoscopy is the primary method for diagnosing colonic diseases and detecting precancerous lesions, thereby playing a crucial role in the prevention of colorectal cancer. However, the scientific quality and reliability of these videos require systematic assessment. In addition, the scientific quality of Turkish colonoscopy videos on YouTube remains unexplored. This study used internationally recognized scoring systems to assess the scientific quality of Turkish colonoscopy-related videos on YouTube. This cross-sectional study analyzed 156 Turkish-language YouTube videos on colonoscopy, with the inclusion criteria requiring a video duration between 30 seconds and 60 minutes. The main outcomes were video quality and information accuracy, which were measured using the *Journal of the American Medical Association* (JAMA), modified DISCERN (mDISCERN) score, colonoscopy data quality (C-DQS), and Global Quality Score (GQS) tools. The study included 156 videos that fulfilled the inclusion criteria. According to the mDISCERN score, 24.4%, 59%, and 16.7% of the videos were of poor, fair, and good quality, respectively. Only 12.8% of the videos met the JAMA’s quality criteria. The overall average score for each video source was low, with an average C-DQS score of 7.1/40. The JAMA, C-DQS, GQS, and mDISCERN scores for the videos designed to educate healthcare professionals were higher than those for videos designed for patient information or general culture/patient experience (*P* < .001 for each). Significant correlations were found between the JAMA score, mDISCERN score, GQS, and C-DQS (*P* < .001). Four independent quality assessment scales were used to evaluate Turkish-language YouTube videos on colonoscopy, with the results indicating suboptimal to moderate information quality. Online platforms, including YouTube, should implement strict quality control measures to ensure the accuracy of health-related videos.

## 1. Introduction

Colorectal cancer (CRC) is the third most prevalent cancer worldwide, accounting for approximately 10% of all cancer cases. In 2020, over 1.9 million new CRC cases and 930,000 CRC-related deaths occurred.^[1]^ CRC is the second leading cause of cancer-related death.^[2]^ According to the Unified Database of Türkiye, CRC is the third leading cancer type in Turkey in both men and women, with approximately 20,000 people diagnosed with colon cancer annually. Colonoscopy is extensively used to diagnose and treat colonic diseases. When the stages of colon cancer are examined, 20.9% of the cases are in the distant stage.^[3]^ As CRC screening expands worldwide, the number of required colonoscopies is also increasing.

Colonoscopy requires intensive bowel preparation, which can trigger anxiety and stress. Anticipatory anxiety about a cancer diagnosis and procedure-related anxiety hinder patients from attending colonoscopy appointments. Failure to undergo a scheduled colonoscopy translates into an increased risk of advanced-stage CRC and death caused by CRC.^[4]^ Previous studies have indicated that showing an information video to patients on the day of their colonoscopy could help curb their anxiety.^[4–6]^ Thus, utilizing improved pre-procedure information in the form of a video has been suggested as a promising intervention for improving adherence to colonoscopy schedules. YouTube is the second-most-visited website worldwide, with >2 billion logged-in users. It serves as a major source of online video information in Turkey, with approximately 57.9 million logged-in users in 2023; 39.5% of Turkish-language people who accessed the Internet searched for information related to health problems and products.^[7]^ Regardless of the communication channel used, the content can influence an individual’s decisions regarding health, including lifestyle changes. However, online resources do not undergo adequate quality control measures,^[8]^ and 86% of health seekers are skeptical about the reliability of online health-related information, whereas 44% believe that these videos are only partially accurate.^[9]^

Patients may turn to the Internet for additional information and to alleviate their anxiety instead of contacting healthcare professionals. However, the educational quality of YouTube videos on various diseases and procedures varies.^[10–14]^ The quality of information on colonoscopy disseminated by YouTube videos has been reported to be suboptimal.^[15–18]^ Therefore, the quality of content available to millions of users on websites such as YouTube should be assessed.

In the Turkish-language context, there is a significant need to produce and share high-quality, reliable, and comprehensible videos of various medical procedures, including ankle fractures, frozen shoulder exercises, and treatment of gingival recession.^[19–21]^ However, to our knowledge, no study has evaluated the scientific quality of Turkish-language colonoscopy videos on YouTube. This study used internationally accepted scoring systems to assess the scientific quality of colonoscopy videos available on YouTube.

## 2. Methods

### 2.1. Search strategy and video selection

This cross-sectional study, conducted from October to 23 to 30, 2024, used Google Trends (Google LLC, Mountain View; https://trends.google.com), a complementary tool that analyzes the popularity of Google search terms to identify search patterns. The term “colonoscopy” was entered into the search box, and the most relevant keywords were retrieved by adjusting the search settings across all categories, including Google Web and YouTube, in the past 5 years (2020–2024). Following the consensus of 2 gastroenterologists, the 4 most popular keywords were selected: “colonoscopy,” “what is colonoscopy,” “how is colonoscopy done,” and “how is colonoscopy preparation diet done?” To mitigate the potential bias from personalized algorithms, new Google and YouTube accounts were created. An online search was performed for these keywords directly on YouTube (http://www.youtube.com) following Turkey’s standard search preference. Google Trends was used solely to identify popular search terms, and actual video search and selection were conducted exclusively on YouTube.

The default search preference of the website, that is, “sort videos by relevance,” was used while disabling all cookies and disregarding advertisements. YouTube provides a continuous list of 25 videos per page as search results. As 95% of online searchers proceed only to the first 3 or 4 pages, we analyzed the top 40 videos for each keyword in the search results.

For this study, 227 videos identified from the search results were saved to a personal YouTube playlist for detailed examination. All preliminary investigations were independently performed by 2 experienced gastroenterologists.

### 2.2. Inclusion and exclusion criteria

Only Turkish articles were included in this study, regardless of the geographic location of upload. The inclusion criteria were as follows: video content available in Turkish, primary subject, and video duration between 30 and 60 minutes. Duplicates, pediatric colonoscopy, virtual colonoscopy, irrelevant videos (e.g., nonmedical content), silent videos, and videos from other social media platforms (e.g., TikTok, Instagram, and X) were excluded from the study.

### 2.3. Data extraction

The subsequent phase of the study was conducted by 2 gastroenterologists who were blinded to each other’s involvement in the study. Two gastroenterologists independently recorded metrics, including origin, source, number of video source subscribers, content category, narrator, upload date, duration, total views, likes, and scores for each rating. The video age (defined as the number of days from the upload date until October 30, 2024) and view rate (calculated as the total number of views divided by the video age) were calculated. Subsequently, the data were subjected to comparative analysis. The videos with discrepant scores were reevaluated. The final scores were derived by averaging the assessments and documented only upon mutual agreement between the 2 gastroenterologists, ensuring a consensus on clinical reasonableness. During the initial days of this study, which spanned approximately days, the video source, presenter’s specialty, video duration, video content, and rating scale were recorded. On the final day, the duration of the video recording, video age, number of views, view rate, number of likes, and number of subscribers were recorded. The videos were categorized into 5 subgroups based on their sources: private hospitals, medical doctors, academic institutions, foundations or associations, health channels/websites, and health-related television broadcasts. The videos were divided into 3 subgroups according to their content: patient information, healthcare professionals’ information, and general culture or patient experiences. The videos were further divided into 3 subgroups according to the people who prepared and presented them: gastroenterology physicians, general surgery physicians, and those who did not perform endoscopy (endoscopy nurses, physicians who did not perform endoscopy, and patients).

### 2.4. Quality assessment tools

Video content was measured using the modified DISCERN (mDISCERN), *Journal of the American Medical Association* (JAMA), Global Quality Score (GQS), and Colonoscopy Data Quality Score (C-DQS). The original DISCERN scoring method, which includes 15 questions, was used to assess the quality of the health information provided to patients. The study utilized the mDISCERN tool, comprising 5 yes/no questions, with each yes response assigned 1 point, yielding a maximum score of 5. The questions assessed the following criteria: Is the video content clear, concise, and comprehensible? Does the video cite authoritative and reliable sources? Is the information balanced and free of bias? Does the video provide supplementary references or resources for patients? And, does the content acknowledge and address the areas of controversy or uncertainty? In the DISCERN scoring, videos with scores >3 were considered high quality and contained useful information for the patient. Videos with a score of 3 were considered of medium quality and required additional information sources. Videos scoring <3 were considered low quality and should not be used by patients.^[22]^

The quality of the information obtained from health-related websites was assessed using the JAMA scoring system. The quality scale includes 4 criteria: authorship, citations, explanations, and timeliness. Each item was evaluated using a score of 0 (does not meet the required criteria) or 1 (meets the required criteria). The lowest and highest scores on these scales were 0 and 4, respectively. Higher scores indicate an increase in the quality of information being assessed.^[23]^ The C-DQS scoring system was created using a colonoscopy training video from the American Society for Gastrointestinal Endoscopy (ASGE) website as the reference standard. Video quality was compared with that of a colonoscopy training video on the ASGE website. The videos were graded with an initial score of 0 and a total score of 10 to 40 points (Table 1). Points were deducted for any misleading information such as “colonoscopy increased the risk of colon cancer” and “colonoscopy is a useless procedure.” Points were awarded for correct information covering all relevant topics as outlined in the ASGE website’s educational videos.^[24]^

**Table 1:** Checklist for the assessment of colonoscopy data quality score.

Defines colonoscopy (1 point) – e.g., colonoscopy is the procedure to look at the large bowel, and it is a safe and effective procedure
Mentions that colonoscopy is currently the best screening method for preventing colon cancer (1 point)
States the prevalence of screening colonoscopy (1 point) – e.g., the number of colonoscopies performed: 14.2 million per year in the USA
Defines a colonoscope (1 point) – it is a flexible tube with a small camera and light at the end, which allows the examination of the colon
Indications for colonoscopy as a diagnostic and therapeutic procedure (1 point each, maximum 5 points)
Colon cancer
Polyps
Hemorrhoids
IBD
Large intestinal disorders
Gives an estimate of lifetime risk of colon cancer (1 point) – e.g., every individual has a 6% risk of developing colon cancer over a lifetime
Mentions that colonoscopy helps to prevent cancer by removing polyps before they turn into cancer (1 point)
Mentions that colonoscopy is the only test that can find and remove polyps at the same time among the various colon cancer screening tests (1 point)
Expectations before the procedure
Mentions that the patient will be given written preparation instructions before the procedure (1 point)
Describes bowel preparation regimen (1 point) – e.g., cleansing begins before the day of the examination, and there are different types of bowel preps
Mentions that the patient should take a clear-liquid diet 1 day before the procedure (1 point)
Emphasizes the importance of good bowel prep for better colon visualization and to avoid cancellation (1 point) – e.g., proper bowel prep means the quicker, safer, more comfortable, and more effective procedure
Recommends no eating or drinking 6 h before the procedure (1 point)
Describes that the doctor will advise the withholding of certain medications before the procedure (1 point)
Encourages patients to ask a question to the medical team if needed (1 point)
Expectation during the procedure
Mentions that informed consent will be obtained after explaining the risks, benefits, and alternatives (1 point)
Mentions that the procedure will be performed under sedation (1 point)
Mentions that IV access will be obtained for sedation (1 point)
Describes the procedure in general (1 point) – e.g., colonoscopy is performed in a procedure room equipped with a colonoscope and a TV screen to see images of the colon
Describes colonoscope and how it works (1 point) – e.g., a flexible tube with a camera and light source at the end with a channel to pass instruments for biopsy. The scope has dials to maneuver in a different direction. The scope will be passed to the cecum
Describes what the endoscopist looks for during the procedure (1 point) – polyps, source of bleeding, inflammation, etc.
Mentions that the duration of colonoscopy is usually less than an hour (1 point)
Mentions that colonoscopy is a well-tolerated procedure (1 point)
Describes complications of the procedure (1 point each, maximum of 4 points)
Perforation
Bleeding
Missing polyp or lesion
Adverse drug reaction
Defines polyp and/or types of polyp (1 point)
Expectation after the procedure
Mentions that the patient will be closely watched for 30 min after the procedure (1 point)
Mentions that the patient will be made aware of preliminary results, new medications, and follow-up plan (1 point)
Mentions that mild bloating and gas are expected after the procedure (1 point)
Mentions what is the biopsy and states that biopsy results can take several days and how to follow up (1 point)
Mentions that the patient should not go to work or drive or handle heavy machinery on the same day (1 point)
Mentions that the patient will need somebody to drive him/her back home (1 point)
Mentions that the patient can return to normal activity the next day following the procedure (1 point)
Advises the patient to call a doctor in case of abdominal pain, bleeding, or fever (1 point)
Negative points
Any false information (not limited to the following) (−1 point each)
Colonoscopy is a useless procedure
Colonoscopy increases the risk of colon cancer
Colonoscopy does not prevent colon cancer
Colonoscopy does not have any supporting scientific evidence
Colonoscopy should not be performed in asymptomatic patients
Colonoscopy is a high-risk procedure with high mortality and complication rates
Colonoscopy is an expensive procedure
Colonoscopy is performed without sedation and is very uncomfortable
Bowel preparation is not needed before colonoscopy
Colonoscopy is solely a diagnostic procedure
Score (maximum 40 points)

IBD = inflammatory bowel disease, IV = intravenous.

### 2.5. Ethical considerations

This study was exempt from ethics committee approval because it only analyzed publicly available YouTube data and did not involve human or animal subjects. This is consistent with the standard practices for observational studies of publicly available social media content. This study did not reproduce, redistribute, or modify any video content. The analysis was limited to publicly available metadata and qualitative assessment of video content, which is consistent with fair use principles for academic research. This study followed the Strengthening the Reporting of Observational Studies in Epidemiology Guidelines.

### 2.6. Statistical analysis

Statistical analyses were performed using IBM SPSS Statistics version 22.0 (International Business Machines Corporation, Armonk). The normality of the continuous data was assessed by examining the skewness and kurtosis values using the Kolmogorov–Smirnov test. Continuous data with a non-normal distribution were compared between groups using the Kruskal–Wallis test, and post hoc Mann–Whitney *U* tests were used for pairwise comparisons. The threshold for statistical significance was set at *P* < .05. Correlations between continuous variables were evaluated using the Spearman rank correlation coefficient. For categorical data, group comparisons were performed using the Pearson chi-square test, and a *z*-test was used for subgroup analyses, with *P* < .05 considered statistically significant.

## 3. Results

**Figure 1.**
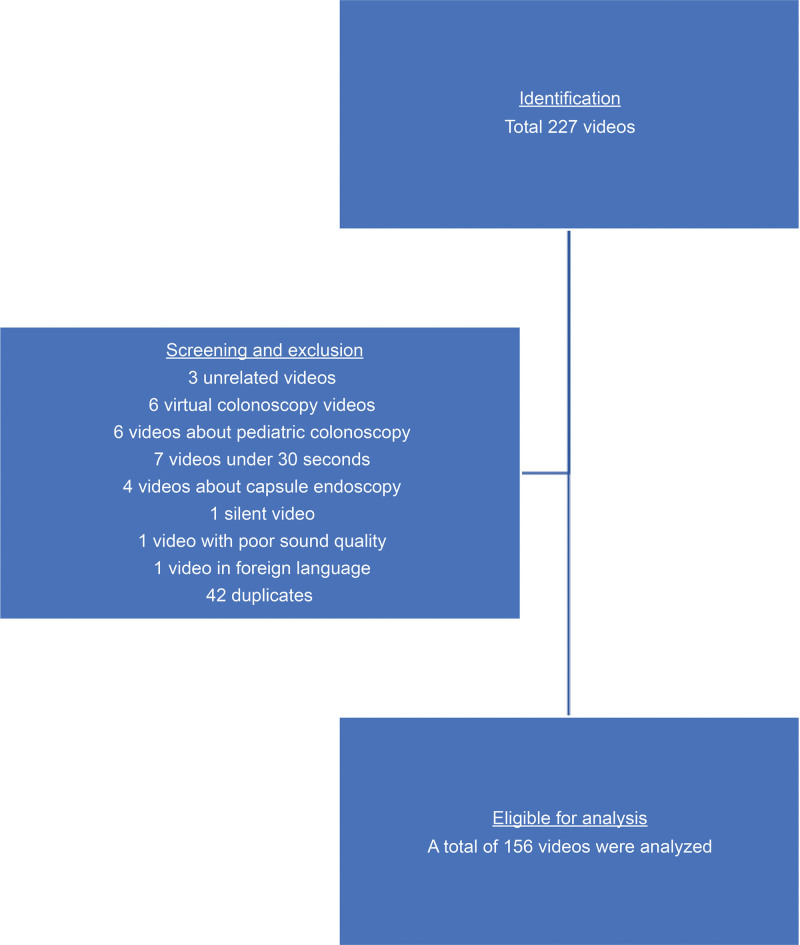
Flowchart of video screening and selection for analysis.

In this study, 227 videos were analyzed. After removing videos that met the exclusion criteria and combining repetitive videos in the YouTube library, 156 videos were analyzed by 2 gastroenterologists who were blinded to the data. No misleading videos were found in any of the searches (Fig. 1).

The average duration of the videos was 3 minutes 52 seconds, and the average number of views was 35,995. According to the mDISCERN classification, 24.4%, 59%, and 16.7% of the videos were of poor, fair, and good quality, respectively. For the JAMA score, 12.8% of the videos met the quality criteria when the cutoff was set at ≥3. The overall average score for each video source was low, with an average C-DQS score of 7.1/40. None of the 156 videos contained unhelpful information. The descriptive characteristics are presented in Tables 2 and 3, respectively.

**Table 2:** Descriptive statistics of colonoscopy videos on YouTube.

	Mean ± SD	(Min–max)
Age (mo)	49.7 ± 31.2	(26.5–67.5)
Number of total views	35.9951 ± 92.1817	(604–29,456)
View ratio (number of views per day)	25.974604 ± 60.2472517	(0.45734172–20.1366667)
Duration (s)	232.8 ± 375.4	(65–221)
Number of likes	139.9 ± 331.2	(4–96.5)
Number of subscribers	82,649.3 ± 680,407.1	(799–23,500)
C-DQS	7.1 ± 4.4	(4–9)

C-DQS = Colonoscopy Data Quality Score, SD = standard deviation.

**Table 3:** Source, creator, and presenter, content, and scores of colonoscopy-related videos.

		N	%
Sources	Medical doctors	61	39.10
	Private hospitals	58	37.20
	Health channels/websites	23	14.70
	Academic institutions/foundations-associations	10	6.40
	Health-related broadcasts of television channels	4	2.60
Prepared and presented	Gastroenterology physicians	87	55.80
	General surgery physicians	61	39.10
	People who do not perform endoscopy (endoscopy nurse, physician who does not perform endoscopy, patient)	8	5.10
Video content	Information for patients	120	76.90
	Education of healthcare professionals	13	8.30
	General culture/patient experience videos	23	14.70
JAMA	1	15	9.60
	2	121	77.60
	3	20	12.80
GQS	1	14	9.00
	2	47	30.10
	3	60	38.50
	4	21	13.50
	5	14	9.00
mDISCERN	1	11	7.10
	2	27	17.30
	3	92	59.00
	4	26	16.70

GQS = Global Quality Score, JAMA = *Journal of the American Medical Association*, mDISCERN = modified DISCERN.

The most common video source was content produced by physicians (39.1%). The median number of views, likes, and duration was 2542, 20, and 2 minutes 9 seconds, respectively. Among the sources, the videos published by academic institutions, foundations, and associations had higher JAMA score, C-DQS, and GQS values than those from other sources (*P* = .003, *P* = .007, and *P* = .035, respectively), and the mDISCERN quality scores were not significantly different (*P* = .145). The median number of subscribers to the TV health program was 255,000, which is the highest among all groups (*P* < .001).

The most popular content was informative videos for patients (76.9%). The JAMA, C-DQS, GQS, and mDISCERN scores of the content prepared for the education of healthcare professionals were higher than those of the other videos (*P* < .001 for each). The duration of the videos prepared for the education of healthcare professionals was longer than that of the others (*P* < .001).

Among those who prepared and presented the videos, 55.8% were gastroenterologists, and 94.9% were physicians who performed endoscopy. Videos prepared and presented by those who did not perform endoscopy (endoscopy nurses [n = 8], physicians who did not perform endoscopy [n = 1], and patients [patients who did not undergo endoscopy, n = 8; patients who underwent endoscopy, n = 1]) had a higher number of views, view rates, likes, and subscribers (*P* = .031, *P* = .008, *P* = .038, and *P* = .011, respectively).

Significant correlations were found between the JAMA score, mDISCERN score, GQS, and C-DQS (*P* < .001). Moreover, the number of likes, views, subscribers, and viewership was significantly correlated (*P* < .001). A high level of positive and significant correlations was found between GQS and both the mDISCERN and C-DQS values (*r* = .767 and *r* = .792, respectively). The correlations between the mDISCERN score, JAMA score, GQS, C-DQS, number of likes, and viewing rates are shown in Table 4.

**Table 4:** Correlation between DISCERN score, JAMA score, GQS, and C-DQS and number of total views, view ratio, number of likes, and number of subscribers.

	Number of total views	View ratio (number of views per day)	Number of likes	Number of subscribers	JAMA	GQS	mDISCERN	C-DQS
JAMA	*r* = .041	*r* = .056	*r* = .056	*r* = .007	1.000	*r* = .550**	*r* = .618**	*r* = .470**
	*P* = .613	*P* = .487	*P* = .484	*P* = .935	–	*P* < .001	*P* < .001	*P* < .001
GQS	*r* = .270**	*r* = .261**	*r* = .245**	*r* = .079	*r* = .550**	1.000	*r* = .767**	*r* = .792**
	*P* = .001	*P* = .001	*P* = .002	*P* = .325	*P* < .001	–	*P* < .001	*P* < .001
mDISCERN	*r* = .187*	*r* = .174*	*r* = .170*	*r* = .084	*r* = .618**	*r* = .767**	1.000	*r* = .629**
	*P* = .020	*P* = .030	*P* = .034	*P* = .297	*P* < .001	*P* < .001	–	*P* < .001
C-DQS	*r* = .170*	*r* = .194*	*r* = .206**	*r* = .037	*r* = .470**	*r* = .792**	*r* = .629**	1.000
	*P* = .034	*P* = .015	*P* = .010	*P* = .648	*P* < .001	*P* < .001	*P* < .001	–

C-DQS = Colonoscopy Data Quality Score, GQS = Global Quality Score, JAMA = *Journal of the American Medical Association*, mDISCERN = modified DISCERN.

* Correlation is significant at the .05 level (two-tailed).

** Correlation is significant at the .01 level (two-tailed).

## 4. Discussion

In this cross-sectional study, the scientific quality of colonoscopy-related YouTube videos published in Turkish was assessed using internationally accepted scoring systems and was found to be low to moderate. To our knowledge, this study is the first to jointly assess the JAMA, mDISCERN, C-DQS, and GQS. In the first known publication that used YouTube as a source of content, Gupta et al evaluated colonoscopy quality but did not use quality scales. In a subsequent colonoscopy quality study, Radadiya et al used the GQS and C-DQS.^18^ Our findings are consistent with those of other studies indicating that the educational quality of YouTube videos concerning erectile dysfunction, bladder cancer, and chemotherapy is generally low to medium.^[11,13,25]^ In addition, most other publications were of low quality.^[10,14,26]^ Only 1 study reported YouTube content with good scientific quality.^17^

Furthermore, when we categorized the videos according to their sources, we found that videos prepared by academic institutions, foundations, or associations had the best quality scores, as reported previously.^[27–29]^ The mDISCERN quality scores were also high; however, the difference was not statistically significant. This may be due to the subjective nature of the mDISCERN quality scores. This resource group also had the lowest number of subscribers, likely because videos prepared by academic institutions, foundations, or associations use academic jargon that the public cannot understand. TV health programs had the highest number of subscribers. This may be related to the fact that professionals are invited to TV health programs and people call to update the information and prepare for the program. A previous study showed that the live televised broadcast of the pioneering American broadcast journalist Katie Couric’s colonoscopy significantly increased screening colonoscopy rates among the general population.^[30]^ To reach a wider audience and counterbalance existing misinformation, resources should be made available on other well-known platforms, especially YouTube, and on channels with many subscribers.

Regarding video content, the quality of the content for the education of healthcare professionals was the highest across all scoring systems, and the video duration was the longest. However, a significant correlation between video content and duration was not found in all previous studies.^[31,32]^ Although some disagreement exists among experts regarding the ideal length of a video, a general recommendation is to keep the length of educational videos short, usually between 5 and 20 minutes, to ensure production efficiency and viewer engagement.^[33,34]^ Videos with the highest viewership according to YouTube search algorithms are not necessarily the best in terms of information accuracy or comprehensibility. The need for video uploaders to provide quality information at optimal times must be emphasized.

In this study, the number of views, ratings, likes, and subscribers was higher for those who did not perform endoscopy (endoscopy nurses, physicians who did not perform endoscopy, and patients) than for those who did. Endoscopy nurses accurately and clearly explained how to prepare for the colonoscopy and diet. Anyone deciding to undergo colonoscopy is likely to carefully watch the video until the end and, if necessary, repeatedly, which may make their videos stand out in the search algorithms.

In contrast to the results of other studies on rheumatoid arthritis and dialysis, all videos in our study contained useful information,^[35,36]^ likely because only 1 video was a patient experience video and the rest were produced by healthcare professionals. However, the scientific quality of the included studies was low to moderate. The content published by healthcare professionals may still be of inadequate or inconsistent quality.^[37]^ Notably, several challenges are inherent in the development of educational videos, such as the cost associated with producing high-quality videos,^[38]^ the requirement of significant spare time (typically limited among academic clinicians),^[39]^ and the rigidity of video as a medium that is difficult to revise. An erectile dysfunction study by Fode et al included no patient experience videos.^11^ While one should avoid oversimplifying and stereotyping ethnic groups, the importance of cultural mediation in health-seeking behavior and diagnosis or treatment perception should be acknowledged. A recent study explored Turkish-language clients’ unique views on health and well-being. Divergent cultural conceptions of health – individualistic/biomedical in the Dutch context versus collectivistic/socio-ecological in the Turkish-language context – create a fundamental dissonance in how attention-deficit/hyperactivity disorder diagnosis and depression treatment are perceived.^[40]^ In our study, the use of experience videos could be attributed to only 1 reason: those who currently undergo colonoscopy in the Turkish-language population want to hide their experience; those who undergo colonoscopy are generally older and may not have a YouTube channel, owing to their late introduction to the Internet; and the majority of YouTubers are young, and the need for colonoscopy is generally low among young people. However, over time, we can expect an increase in patient experience videos on this subject as the need for colonoscopy increases among digital content producers with many followers.

This study had some limitations. First, it employed a cross-sectional design and may not reflect the evolving structure of websites. Second, it focuses solely on Turkish videos available to viewers in Türkiye. Third, the videos were ranked in order of popularity, and the evaluation was performed on only 1 day. However, YouTube is a rapidly evolving dynamic website, and the order of video popularity can change on a daily basis. We queried the search engine using a specific set of search terms for a single day, and it is possible that more extensive materials have been published since then. Moreover, the video age, number of views, view rate, number of likes, and number of subscribers can change daily. Fourth, although JAMA, mDISCERN, GQS, and C-DQS are reliable scoring scales, they have not yet been validated. Fifth, at the time of this study, YouTube did not publicly display dislikes. Therefore, the video power index ([number of likes/dislikes + number of likes] × 100) could not be calculated. We also ignored comments, as many of the videos had no comments or the comments section was closed. Sixth, our search was limited to YouTube; it did not consider TikTok (TikTok Inc., Culver City) or Instagram (Meta Platforms, Menlo Park), which are more personal instructional videos. Finally, our study included individuals with different medical backgrounds, making it difficult to assess how individuals with nonmedical backgrounds or poor health literacy rated the videos. Future studies should include raters without a medical background to better understand their perspectives.

In conclusion, this study represents one of the first comprehensive evaluations of the scientific quality of Turkish colonoscopy videos, raising critical awareness of the reliability of medical content on social media platforms. Poor-quality videos may contribute to patient misinformation and exacerbate fears about colonoscopy, potentially undermining the effectiveness of screening programs. Future research should examine different platforms, patient populations, and content types to explore strategies for improving digital health literacy. Healthcare institutions should promote the dissemination of verified and standardized content on platforms such as YouTube and encourage expert-reviewed educational videos to enhance public understanding and trust.

## Acknowledgments

We sincerely thank gastroenterologist Ümit Çelik for his statistical assistance and help in preparing the tables.

## Author contributions

**Conceptualization:** Ibrahimhalil Dusunceli.

**Methodology:** Ibrahimhalil Dusunceli, Zeynep Gök Sargin.

**Supervision:** Ibrahimhalil Dusunceli.

**Formal analysis:** Zeynep Gök Sargin, Fatih Sargin.

**Investigation:** Zeynep Gök Sargin.

**Visualization:** Zeynep Gök Sargin.

**Data curation:** Fatih Sargin.

**Resources:** Fatih Sargin.

**Validation:** Fatih Sargin.

**Writing – review & editing:** Ibrahimhalil Dusunceli, Fatih Sargin.

**Writing – original draft:** Zeynep Gök Sargin.
